# Impact of combined skeletal muscle index, subcutaneous fat index, and visceral fat index on prognosis in non-metastatic non-small cell lung cancer

**DOI:** 10.1186/s12890-026-04235-w

**Published:** 2026-03-12

**Authors:** Fahui Chen, Zhihui Shi, Hua Bai, Hongtao Lei, Hongjiang Pu, Yang Yang, Yongmei Wu, Ying Zhao, Xin Ning, Guanghong Yan, Mengmei Liu, Yani Li, Sifan Duan, Hanqun Liu, Chang Shu, Lu Lu, Wenjing Xia, Xuewen Zhang, Zhenhui Li, Dingyun You

**Affiliations:** 1 Yunnan Provincial Key Laboratory of Public Health and Biosafety & School of Public Health, Kunming Medical University, Kunming, Yunnan, 650500 People’s Republic of China; 2 Department of Radiology, Yunnan Cancer Hospital, the Third Affiliated Hospital of Kunming Medical University, Yunnan Cancer Center, Kunming, 650118 China; 3 Department of Oncology, Dazhou Central Hospital, Dazhou, 635000 Sichuan China; 4 Department of Colorectal Surgery, Yunnan Cancer Hospital, the Third Affiliated Hospital of Kunming Medical University, Yunnan Cancer Center, Kunming, 650118 China; 5 School of Nursing, Kunming Medical University, Kunming, 650500 People’s Republic of China; 6 Department of Radiology, Dali Bai Autonomous Prefecture Chinese Medicine Hospital, Dali, 671000 China

**Keywords:** Non-small cell lung cancer, Body composition, Computed tomography, Skeletal Muscle, Prognosis

## Abstract

**Background:**

The combined prognostic role of skeletal muscle index (SMI), subcutaneous fat index (SFI), and visceral fat index (VFI) in non-metastatic non-small cell lung cancer (NSCLC) remains unclear.

**Methods:**

Consecutive non-metastatic NSCLC patients who underwent radical pulmonary resection at Yunnan Cancer Hospital from January 2013 to December 2018 were analyzed. Preoperative CT-derived SMI, SFI, and VFI at the third lumbar vertebra level (L3) were stratified into sex-specific high and low groups. A composite index was created based on the count of low-value measures among the three indices. Cox regression evaluated associations with overall survival (OS) and relapse-free survival (RFS).

**Results:**

A total of 1661 patients (mean age 58.9 ± 9.5 years; 911 men [54.8%] and 750 women [45.2%]) were enrolled, with a median follow-up of 73.97 months (95% CI: 72.80–75.10). Low SMI (HR = 1.49, 95% CI: 1.16–1.92, *p* = 0.002), low SFI (HR = 1.54, 95% CI: 1.22–1.94, *p* < 0.001), and low VFI (HR = 1.69, 95% CI: 1.32–2.17, *p* < 0.001) were associated with poorer OS. The composite index demonstrated poorer OS with an increasing number of low indices (SMI, SFI, and VFI) (*p* for trend < 0.001): one-low (HR = 1.50, 95% CI: 1.16–1.94, *p* = 0.002), two-low (HR = 1.71, 95% CI: 1.29–2.28, *p* < 0.001), and all-low (HR = 2.99, 95% CI: 1.89–4.71, *p* < 0.001). Similar trend occurred for RFS (all *p* < 0.05).

**Conclusion:**

Preoperative SMI, SFI, and VFI were independently associated with prognosis in patients with NSCLC, and a composite index integrating these measures may provide valuable complementary information for risk stratification.

**Supplementary Information:**

The online version contains supplementary material available at 10.1186/s12890-026-04235-w.

## Introduction

Among patients with non-small cell lung cancer (NSCLC) undergoing radical resection, approximately 30% to 55% experience disease recurrence or death [1]. Skeletal muscle index (SMI), subcutaneous fat index (SFI), and visceral fat index (VFI) are established body composition metrics with recognized prognostic relevance in cancer populations [2–4]. These indices have also been associated with differential immunotherapy responses and varied clinical outcomes [5, 6]. However, current prognostic studies examining these factors exhibit considerable heterogeneity, and their combined prognostic utility remains insufficiently explored.

Body composition analysis offers a more precise assessment of muscle and adipose tissue distribution than body mass index (BMI) [7–9]. Recent evidence emphasizes the impact of changes in muscle and fat compartments on cancer progression [10, 11]. Computed tomography (CT) has emerged as a reliable imaging modality for quantitative assessment of body composition [12–14]. While many studies have employed chest CT scans to evaluate body composition in relation to NSCLC outcomes [15–20], significant discrepancies exist between measurements obtained at the chest level and those derived from the third lumbar vertebra (L3) level [21].

Notably, L3-level measurements are considered more standardized and representative of whole-body composition, yet their prognostic significance in NSCLC has not been fully established [22]. Furthermore, recent findings indicate functional crosstalk between skeletal muscle and adipose tissue [23], underscoring the importance of evaluating their combined prognostic impact. Therefore, a comprehensive investigation of L3-based SMI, SFI, and VFI—and their integration into a composite index—may enhance risk stratification in patients with resectable NSCLC.

This study aims to assess the prognostic value of preoperative SMI, SFI, and VFI in patients with NSCLC undergoing radical resection, with a focus on their combined impact on survival outcomes.

## Methods

### Participants

**Fig. 1:**
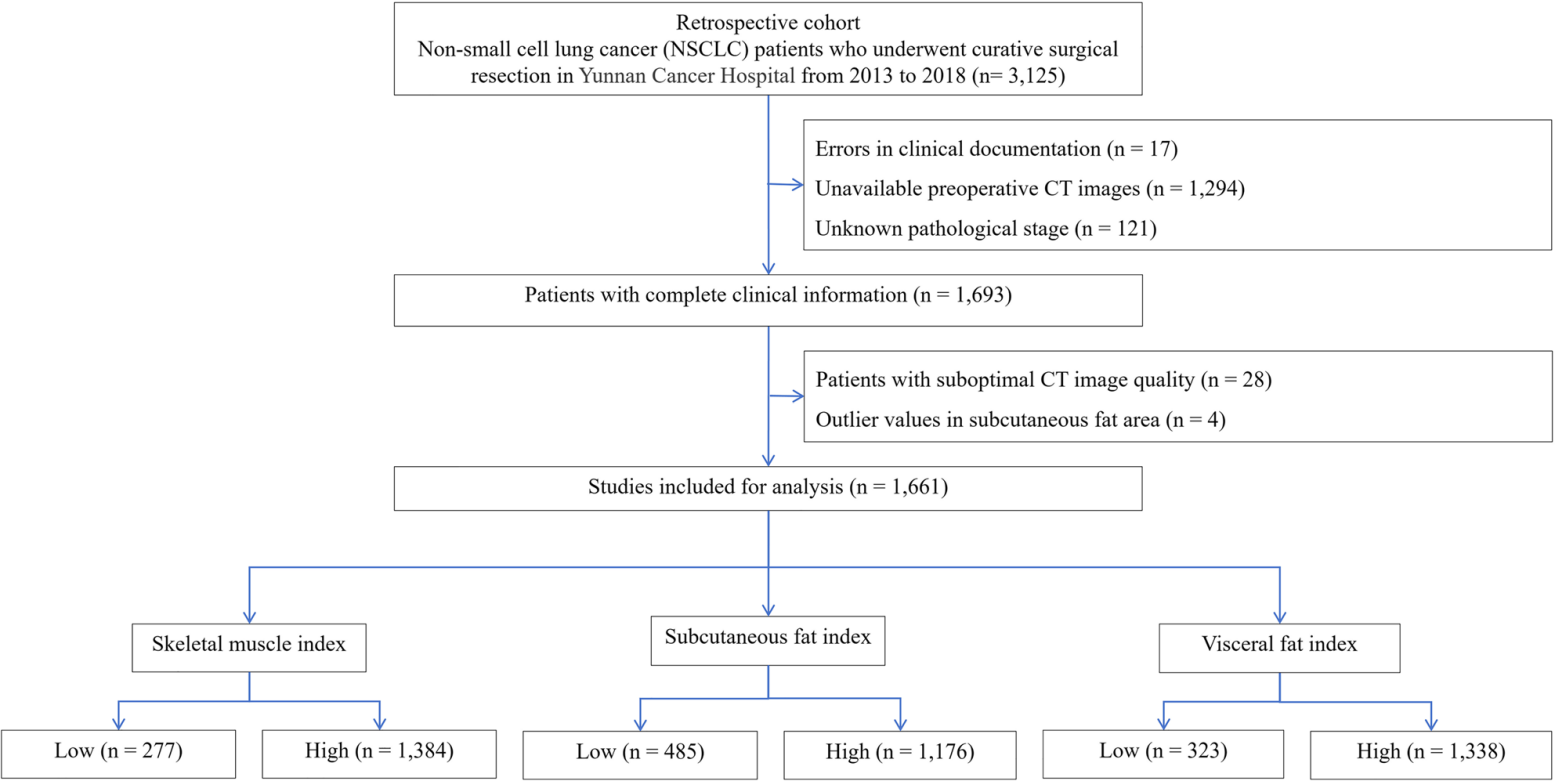
Patient selection flowchart

A total of 3125 consecutive patients with NSCLC who underwent curative-intent surgical resection at Yunnan Cancer Hospital between January 2013 and December 2018 were retrospectively screened. Exclusion criteria included: (1) errors in clinical documentation (*n* = 17); (2) preoperative L3 CT images were unavailable due to hospital changing its imaging storage system, or because patients underwent emergency surgery or were referred from external hospitals and did not undergo preoperative CT scans at our institution (*n* = 1294); (3) unknown pathological stage (*n* = 121); (4) suboptimal CT image quality (*n* = 28); and (5) outlier or unmeasurable subcutaneous fat area (*n* = 4). After applying these criteria, a total of 1661 patients with complete clinical and imaging data were included in the final analysis (Figure 1).

### Quantitative body composition assessment and normalization

A CT-based body composition analysis protocol was implemented, utilizing SliceOmatic software (version 6.0; Tomovision, Montreal, QC, Canada) for quantitative assessment of cross-sectional areas (cm²) of skeletal muscle and adipose tissue at the L3 level on non-contrast CT scans. The Hounsfield unit (HU) thresholds for tissue segmentation were defined as follows: (1) Skeletal muscle area (SMA): -29 to 150 HU, including the psoas major, erector spinae, quadratus lumborum, transversus abdominis, external and internal obliques, and rectus abdominis muscles; (2) Subcutaneous adipose tissue (SAT) : -190 to -30 HU, comprising superficial adipose deposits between the muscular layers and skin in both abdominal and dorsal regions; (3) Visceral adipose tissue (VAT) : -150 to -50 HU, encompassing intra-abdominal fat deposits delineated by the parietal peritoneum or transversalis fascia. The anatomical boundaries of muscle and adipose tissues were precisely delineated using standardized semi-automated segmentation techniques by board-certified radiologists specializing in abdominal imaging, who were blinded to patients’ clinical characteristics and outcomes. SMI, SFI, and VFI (unit: cm²/m²) were calculated by dividing the cross-sectional areas of SMA (cm²), SAT (cm²), and VAT (cm²) by height squared (m²) [2, 24].

### Classification criteria for SMI, SFI, VFI and their composite index

In accordance with established methodologies [25, 26], optimal cutoff values for SMI, SFI, and VFI in relation to OS were determined using the surv_cutpoint function from the “survminer” package in R software, with gender-specific thresholds applied, enabling binary stratification of participants into high versus low value cohorts. The Composite index was generated by summing the number of indices (SMI, SFI, VFI) below their gender-specific cutoffs.

### Outcome definition

The primary endpoint was defined as overall survival (OS), measured from the date of surgery to either death from any cause or the last follow-up. Relapse-free survival (RFS), defined as the time from surgery to locoregional recurrence, distant metastasis, or death from any cause, whichever occurs first.

### Statistical analysis

Baseline characteristics were summarized as numbers (percentages). Categorical variables were compared between groups using χ² tests or Fisher’s exact tests. Kaplan-Meier curves were constructed, and between-group differences were assessed with log-rank tests. Multivariable Cox regression models evaluated the associations between body composition and clinical outcomes, reporting adjusted hazard ratio (HR) with 95% confidence intervals (CI). A sensitivity analysis was conducted following the exclusion of patients with follow-up durations shorter than 6 months. Subgroup analyses were performed stratified by gender, age, pathological stage, tumor location, histological type, adjuvant chemotherapy status, and surgical approach. The subgroup analysis results were visually presented using forest plots generated by the R package “forestploter”. A statistical significance threshold of α = 0.05 was applied for all analyses. All data cleaning and analytical procedures were performed using R statistical software (version 4.4.1).

## Results

### Patient characteristics

Among initially enrolled 3125 patients, 1661 ultimately met the predefined analytical criteria (Figure 1). Comparative baseline data between included and excluded populations are presented in Supplementary Table 1. The study population included 911 men (54.8%) and 750 women (45.2%) (Table 1), with an overall mean age of 58.9 ± 9.5 years (Supplementary Table 1). There were 432 deaths observed during follow-up. Median OS and RFS durations were 73.97 (interquartile range [IQR]: 46.13–90.77) and 70.50 (IQR: 28.63–87.73) months, respectively. Male patients had median SMI, SFI, and VFI measurements of 48.57, 29.74, and 32.26 cm²/m², respectively. The adopted cutoff values for these indices were 41.41, 14.14, and 9.18 cm²/m², correspondingly. Among female patients, the median values for SMI, SFI, and VFI were 39.00, 56.61, and 29.71 cm²/m², respectively. The corresponding cutoff values were established at 33.79, 52.32, and 16.98 cm²/m², respectively.

**Table 1:** Baseline characteristics of body composition profiles in the study cohort

Variable^a^	Total^b^	Skeletal muscle index^b^		*p* ^c^	Subcutaneous fat index^b^		*p* ^c^	Visceral fat index^b^		*p* ^c^
		LowHigh			LowHigh			LowHigh		
Number of patients	1661	277 (16.7)	1384 (83.3)		485 (29.2)	1176 (70.8)		323 (19.4)	1338 (80.6)	
Age, years				< 0.001			0.752			< 0.001
< 60	865 (52.1)	86 (31.0)	779 (56.3)		256 (52.8)	609 (51.8)		201 (62.2)	664 (49.6)	
≥ 60	796 (47.9)	191 (69.0)	605 (43.7)		229 (47.2)	567 (48.2)		122 (37.8)	674 (50.4)	
Sex				0.326			< 0.001			< 0.001
Male	911 (54.8)	144 (52.0)	767 (55.4)		162 (33.4)	749 (63.7)		137 (42.4)	774 (57.8)	
Female	750 (45.2)	133 (48.0)	617 (44.6)		323 (66.6)	427 (36.3)		186 (57.6)	564 (42.2)	
BMI, kg/m^2^				< 0.001			< 0.001			< 0.001
< 18.5	86 ( 5.2)	47 (17.0)	39 ( 2.8)		70 (14.4)	16 ( 1.4)		63 (19.5)	23 ( 1.7)	
18.5–24.9	1264 (76.1)	223 (80.5)	1041 (75.2)		407 (83.9)	857 (72.9)		256 (79.3)	1008 (75.3)	
≥ 25	311 (18.7)	7 ( 2.5)	304 (22.0)		8 ( 1.6)	303 (25.8)		4 ( 1.2)	307 (22.9)	
Smoking				0.729			< 0.001			0.022
Yes	672 (40.5)	109 (39.4)	563 (40.7)		126 (26.0)	546 (46.4)		111 (34.4)	561 (41.9)	
No	970 (58.4)	164 (59.2)	806 (58.2)		357 (73.6)	613 (52.1)		210 (65.0)	760 (56.8)	
Unknown	19 ( 1.1)	4 ( 1.4)	15 ( 1.1)		2 ( 0.4)	17 ( 1.4)		2 ( 0.6)	17 ( 1.3)	
Hypertension				0.008			< 0.001			< 0.001
Yes	226 (13.6)	36 (13.0)	190 (13.7)		40 ( 8.2)	186 (15.8)		15 ( 4.6)	211 (15.8)	
No	1178 (70.9)	181 (65.3)	997 (72.0)		362 (74.6)	816 (69.4)		260 (80.5)	918 (68.6)	
Unknown	257 (15.5)	60 (21.7)	197 (14.2)		83 (17.1)	174 (14.8)		48 (14.9)	209 (15.6)	
Diabetes mellitus				0.012			0.021			0.001
Yes	81 ( 4.9)	14 ( 5.1)	67 ( 4.8)		13 ( 2.7)	68 ( 5.8)		3 ( 0.9)	78 ( 5.8)	
No	1323 (79.7)	204 (73.6)	1119 (80.9)		390 (80.4)	933 (79.3)		272 (84.2)	1051 (78.6)	
Unknown	257 (15.5)	59 (21.3)	198 (14.3)		82 (16.9)	175 (14.9)		48 (14.9)	209 (15.6)	
COPD				0.012			0.518			0.897
Yes	57 ( 3.4)	7 ( 2.5)	50 ( 3.6)		16 ( 3.3)	41 ( 3.5)		12 ( 3.7)	45 ( 3.4)	
No	1346 (81.0)	211 (76.2)	1135 (82.0)		386 (79.6)	960 (81.6)		263 (81.4)	1083 (80.9)	
Unknown	258 (15.5)	59 (21.3)	199 (14.4)		83 (17.1)	175 (14.9)		48 (14.9)	210 (15.7)	
Histologic type				0.626			0.003			0.372
Adenocarcinoma	1283 (77.2)	210 (75.8)	1073 (77.5)		400 (82.5)	883 (75.1)		259 (80.2)	1024 (76.5)	
Squamous cell carcinoma	300 (18.1)	51 (18.4)	249 (18.0)		64 (13.2)	236 (20.1)		51 (15.8)	249 (18.6)	
Other	78 ( 4.7)	16 ( 5.8)	62 ( 4.5)		21 ( 4.3)	57 ( 4.8)		13 ( 4.0)	65 ( 4.9)	
Pathological stage				0.044			0.495			0.085
Ⅰ	959 (57.7)	145 (52.3)	814 (58.8)		287 (59.2)	672 (57.1)		173 (53.6)	786 (58.7)	
Ⅱ	249 (15.0)	54 (19.5)	195 (14.1)		65 (13.4)	184 (15.6)		46 (14.2)	203 (15.2)	
Ⅲ	453 (27.3)	78 (28.2)	375 (27.1)		133 (27.4)	320 (27.2)		104 (32.2)	349 (26.1)	
N stage				0.893			0.424			0.669
< N2	1310 (78.9)	221 (79.8)	1089 (78.7)		386 (79.6)	924 (78.6)		258 (79.9)	1052 (78.6)	
≥N2	297 (17.9)	48 (17.3)	249 (18.0)		80 (16.5)	217 (18.5)		57 (17.6)	240 (17.9)	
Unknown	54 ( 3.3)	8 ( 2.9)	46 ( 3.3)		19 ( 3.9)	35 ( 3.0)		8 ( 2.5)	46 ( 3.4)	
Tumor location				0.637			0.606			0.468
Upper lobe	840 (50.6)	136 (49.1)	704 (50.9)		240 (49.5)	600 (51.0)		157 (48.6)	683 (51.0)	
Non–upper lobe	821 (49.4)	141 (50.9)	680 (49.1)		245 (50.5)	576 (49.0)		166 (51.4)	655 (49.0)	
Preoperative CEA, ng/mL				0.397			0.031			0.008
< 5	1112 (66.9)	179 (64.6)	933 (67.4)		306 (63.1)	806 (68.5)		194 (60.1)	918 (68.6)	
≥ 5	440 (26.5)	75 (27.1)	365 (26.4)		150 (30.9)	290 (24.7)		107 (33.1)	333 (24.9)	
Unknown	109 ( 6.6)	23 ( 8.3)	86 ( 6.2)		29 ( 6.0)	80 ( 6.8)		22 ( 6.8)	87 ( 6.5)	
Chemotherapy				0.151			0.030			0.529
Yes	800 (48.2)	122 (44.0)	678 (49.0)		213 (43.9)	587 (49.9)		150 (46.4)	650 (48.6)	
No	861 (51.8)	155 (56.0)	706 (51.0)		272 (56.1)	589 (50.1)		173 (53.6)	688 (51.4)	
Radiotherapy				0.908			0.279			0.865
Yes	107 ( 6.4)	16 ( 5.8)	91 ( 6.6)		24 ( 4.9)	83 ( 7.1)		18 ( 5.6)	89 ( 6.7)	
No	1542 (92.8)	259 (93.5)	1283 (92.7)		458 (94.4)	1084 (92.2)		303 (93.8)	1239 (92.6)	
Unknown	12 ( 0.7)	2 ( 0.7)	10 ( 0.7)		3 ( 0.6)	9 ( 0.8)		2 ( 0.6)	10 ( 0.7)	

^a^*BMI* Body mass index,* CEA* Carcinoembryonic Antigen,* COPD* Chronic obstructive pulmonary disease

^b^Data are N(%)

^c^*p*, using chi-square test or exact Fisher test

As demonstrated in Table 1, among the baseline characteristics, 277 patients (16.7%) exhibited low SMI, which was significantly associated with advanced age, lower BMI, pathological stage, hypertension, diabetes mellitus, and chronic obstructive pulmonary disease (COPD). Low SFI was identified in 485 patients (29.2%) and was significantly associated with female sex, lower BMI, non-smoking history, adenocarcinoma histology, elevated preoperative carcinoembryonic antigen (CEA) levels, absence of chemotherapy, hypertension, and diabetes mellitus. Low VFI was identified in 323 patients (19.4%) and was significantly associated with younger age, female sex, lower BMI, non-smoking history, elevated CEA levels, hypertension, and diabetes mellitus (all *p* < 0.05).

### Association between individual body composition and clinical outcome

**Fig. 2:**
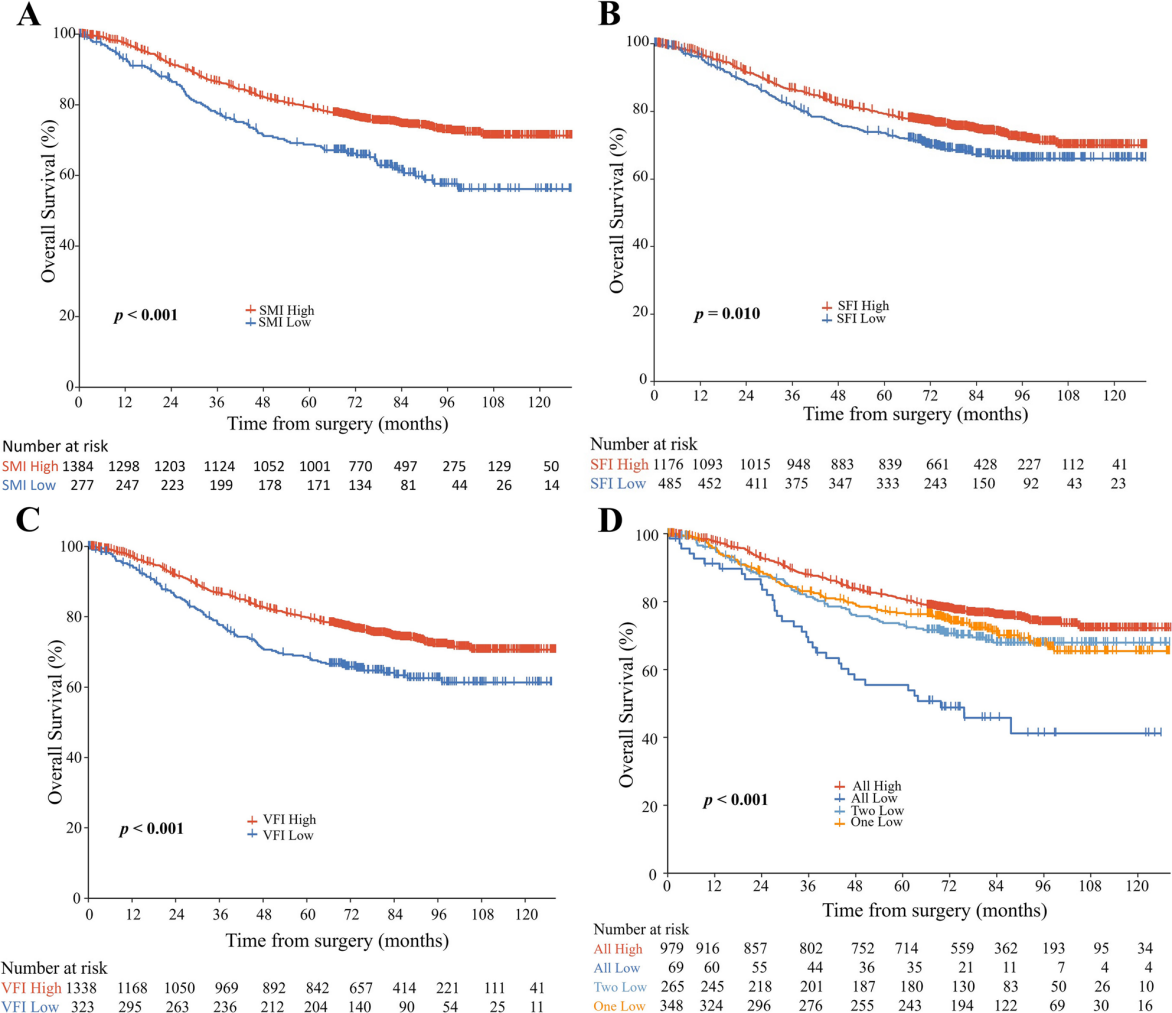
Kaplan-Meier survival curves comparing overall survival among patient groups stratified by skeletal muscle index (SMI) (**A**), subcutaneous fat index (SFI) (**B**), visceral fat index (VFI) (**C**), and their composite index (number of low-value indices: SMI, SFI, and VFI) (**D**). Statistical significance was determined using log-rank tests

**Fig. 3:**
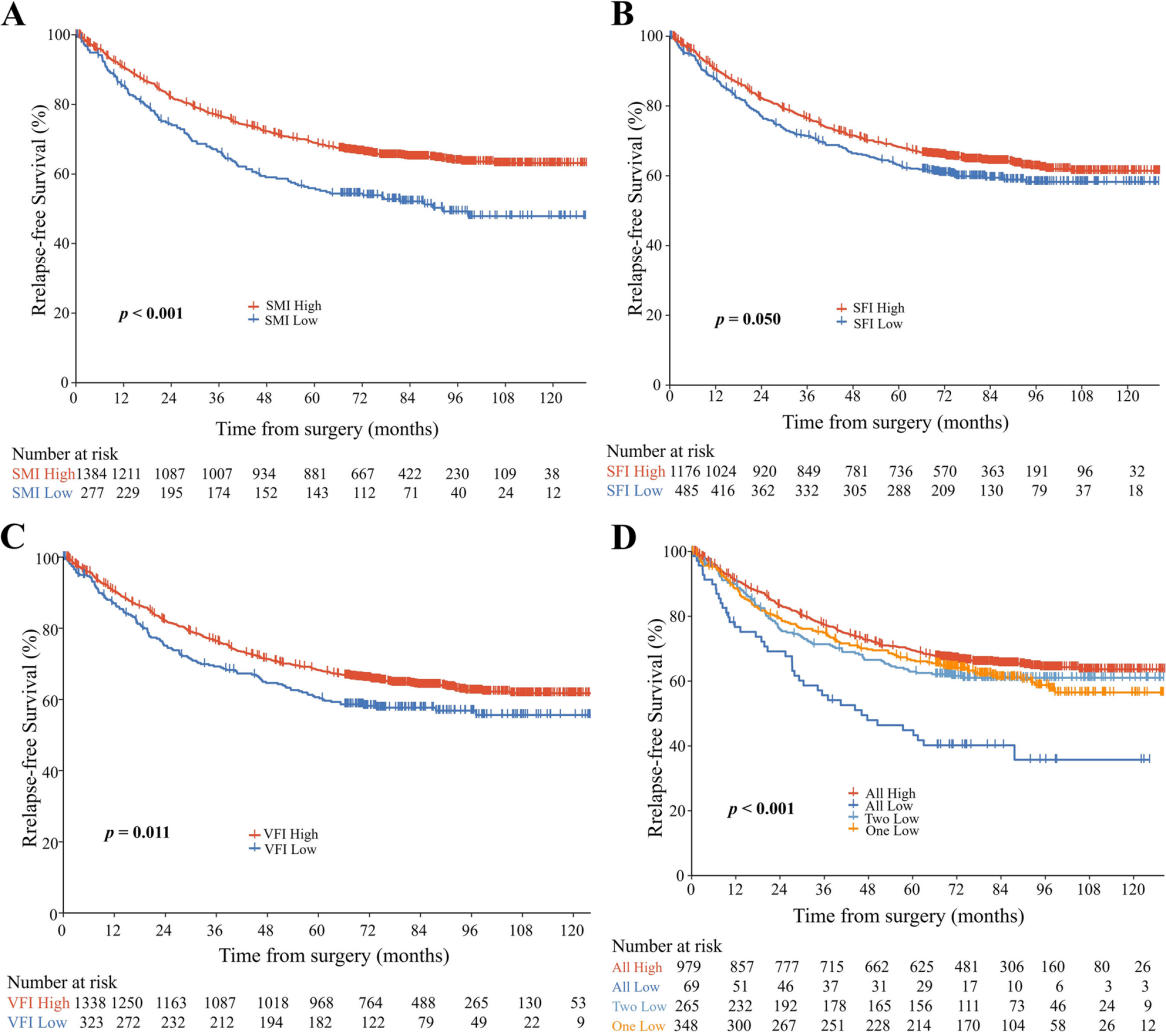
Kaplan-Meier survival curves comparing relapse-free survival among patient groups stratified by skeletal muscle index (SMI) (**A**), subcutaneous fat index (SFI) (**B**), visceral fat index (VFI) (**C**), and their composite index (number of low-value metrics: SMI, SFI, and VFI) (**D**). Statistical significance was determined using log-rank tests

As demonstrated in Figures 2A, B, and C, 5-year OS disadvantage for low vs. high SMI (68.8%, 95% CI [63.3–74.7%] vs. 79.4%, 95% CI [77.2–81.6%], *p* < 0.001); Poorer outcomes with low SFI (73.7% [69.8–77.8%] vs. 79.3% [77.0−81.8%], *p* = 0.010); Similarly, the low VFI group showed reduced 5-year OS relative to the high VFI group (68.7%, 95% CI [63.7–74.1%] vs. (79.8%, 95% CI [77.6–82.1%], *p* < 0.001). Consistent results were observed for SMI and VFI in relation to 5-year RFS (all *p* < 0.05) (Figure 3A and C). No significant difference in 5-year RFS was observed between the high and low SFI groups (*p* = 0.050) (Figure 3B).

The results of the multivariate analysis are presented in Table 2. Low SMI predicted worse OS (HR = 1.49, 95% CI: 1.16–1.92, *p* = 0.002); Low SFI impaired OS (HR = 1.54, 95% CI: 1.22–1.94, *p* < 0.001); Low VFI was associated with increased mortality risk (HR = 1.69, 95% CI: 1.32–2.17, *p* < 0.001). The associations between these three indices and RFS demonstrated similar statistical significance (all *p* < 0.05) (Table 2).

**Table 2:** Multivariable-adjusted analyses of skeletal muscle index, subcutaneous fat index, visceral fat index, and composite index in relation to overall survival and relapse-free survival

Variable^a^	N Patients	Overall Survival			Relapse-free Survival		
		N Events	HR (95% CI)^a^	*p* ^b^	N Events	HR (95% CI)^a^	*p* ^b^
SMI							
High	1384	332	Ref		461	Ref	
Low	277	100	1.49 (1.16–1.92)	0.002	129	1.35 (1.09–1.67)	0.006
SFI							
High	1176	285	Ref		401	Ref	
Low	485	147	1.54 (1.22–1.94)	< 0.001	189	1.39 (1.13–1.70)	0.002
VFI							
High	1338	322	Ref		458	Ref	
Low	323	110	1.69 (1.32–2.17)	< 0.001	132	1.30 (1.04–1.64)	0.021
Composite index*							
All high	979	222	Ref		320	Ref	
One low	348	98	1.50 (1.16–1.94)	0.002	131	1.36 (1.09–1.70)	0.006
Two low	265	77	1.71 (1.29–2.28)	≤ 0.001	98	1.38 (1.07–1.78)	0.013
All low	69	35	2.99 (1.89–4.71)	< 0.001	41	2.13 (1.43–3.16)	< 0.001
* P* for trend				< 0.001			< 0.001

^a^*CI* Confidence interval,* HR* Hazard ratio, *Ref* Reference,* SMI* Skeletal muscle index, *SFI* Subcutaneous fat index, *VFI* Visceral fat index

^b^Multivariate analysis was adjusted for Sex, Age, Smoking history, Hypertension, Diabetes mellitus, Chronic obstructive pulmonary disease, BMI, CEA, N stage, Chemotherapy, Radiotherapy, Pathological stage, Tumor location, Histologic type

*Composite index, number of low values in preoperative SMI, SFI, and VFI

### Body composition composite index as prognostic biomarkers

The worst prognostic outcomes were observed in patients exhibiting low index of all three indices (SMI, SFI, and VFI; all *p* < 0.05) in the combined analysis (Figures 2D and 3D). The 5-year OS was 80% (95% CI: 78.3–83.4%) in the all-high group versus 55.4% (95% CI: 44.6–69.0%), 73.2% (95% CI: 67.9–78.9%), and 76.6% (95% CI: 72.2–81.3%) in groups with all-low, two-low, or one-low, respectively.

Multivariate analysis demonstrated progressively worse OS with an increasing number of low indices (SMI, SFI, or VFI; *p* for trend < 0.001): HR = 2.99 (95% CI: 1.89–4.71; *p* < 0.001) for the all-low group, HR = 1.71 (95% CI: 1.29–2.28; *p* < 0.001) for the two-low group, and HR = 1.50 (95% CI: 1.16–1.94; *p* = 0.002) for the one-low group, vs. all-high group, and a consistent pattern was also observed in RFS (all *p* < 0.05) (Table 2). The all-low group had the worst 3-year and 5-year survival outcomes across all pathological stages except for stage IIIB (Supplementary Fig. 1).

### Subgroup and sensitivity analyses

**Fig. 4:**
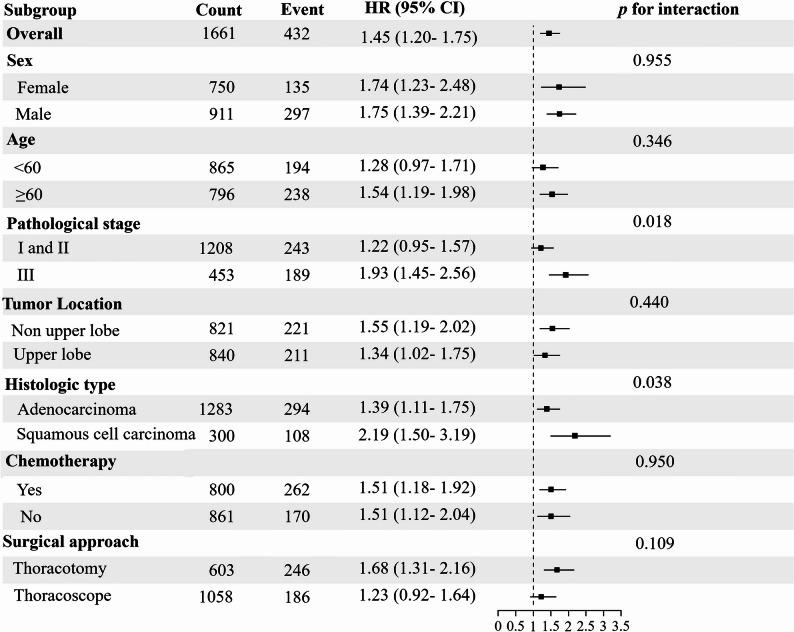
Forest plots present the analysis results of body composition abnormality (defined by low skeletal muscle index [SMI], subcutaneous fat index [SFI], or visceral fat index [VFI]) subgroups stratified by clinicopathological variables

Sensitivity analyses confirmed the robustness of the primary findings, with all associations remaining statistically significant (*p* < 0.05; Supplementary Table 2). Subgroup analyses showed that SMI, SFI, or VFI were consistently associated with shorter OS across most strata (Figure 4). However, this association was not statistically significant in patients younger than 60 years, those with pathological stage I/II disease, or those who underwent thoracoscopic surgery—although the directional trends remained consistent with the overall cohort. Furthermore, significant interactions were observed between body composition indices and both pathological stage (*p* = 0.018) and histological type (*p* = 0.038), suggesting effect modification (Figure 4).

Further stratified analyses showed that a low composite index was associated with poor prognosis in both stages I/II and stage III disease, although the risk patterns differed by stage. In early-stage disease, low SMI and low VFI were associated with increased risk, with the poorest survival observed only when all three indices (SMI, SFI, and VFI) were concurrently reduced, indicative of severe systemic wasting. In advanced-stage disease, low SMI, low SFI, and low VFI were each independent risk factors, with the highest risk seen in patients with concomitant reductions in two indices, whereas further reduction across all three indices did not amplify risk (Supplementary Table 3).

## Discussion

This study developed a novel composite body composition index by integrating SMI, SFI, and VFI based on low-value index counts. Associations were identified between composite indices and clinical prognosis in NSCLC patients. Multivariable analyses showed worsening OS and RFS with increasing numbers of below-threshold body composition indices (SMI, SFI, or VFI). Reduced SMI was independently associated with worse clinical outcomes, consistent with previous studies assessing SMI at alternative anatomical landmarks [16, 18, 27]. A recent systematic review further supports the prognostic relevance of SFI and VFI, demonstrating their associations with poor survival in various cancers [28]. Our findings confirm these associations in NSCLC, aligning with results from multiple independent cohorts [29–31]. Contrary to our results, Barbi et al. reported that high VFI predicted worse OS and RFS [32]. These differences may result from distinct cutoff selection methods (maximal selected rank statistics vs. tertile-based approach) and inherent tumor heterogeneity. Reduced muscle mass may adversely affect functional status and overall health in patients with NSCLC [33].

This study found that patients with concomitant low SMI, SFI, and VFI generally have poorer prognosis, suggesting that concurrent depletion of muscle and fat may constitute a high-risk body composition phenotype. Muscle wasting may reduce the secretion of immunoregulatory myokines, while abnormal fat loss or redistribution may disrupt adipokine signaling and metabolic regulation, thereby impairing immune and metabolic functions [34, 35]. This combined deficiency of muscle and fat may compromise the body’s capacity to adapt to inflammatory and energetic stress, reduce treatment tolerance, and increase the risk of cachexia, ultimately contributing to adverse outcomes [36, 37]. Although these mechanisms are supported by experimental and clinical evidence, their precise pathways require further investigation.

Another key finding of this study was the significant interaction between pathological stage, histologic type, and abnormalities in body composition (all *p* < 0.05). Although body composition was significantly associated with overall survival in the overall cohort, no statistically significant association was observed in the subgroup of patients with stages I/II disease (Figure 4). This difference may primarily be attributed to the clinical characteristic differences between patients at different disease stages. Advanced-stage patients are more likely to experience significant metabolic disturbances and systemic inflammation, which can exacerbate the impact of body composition deterioration on treatment tolerance and survival outcomes [38, 39]. By contrast, prognosis in early-stage disease is more strongly driven by tumor biology and the completeness of surgical resection, potentially attenuating the observable influence of body composition [40]. Notably, even in stages I/II patients, the direction of the hazard ratios was consistent with that of the overall cohort (Figure 4), and mortality risk remained significantly increased in those in the composite index all-low group (Supplementary Table 3), indicating that severe body composition impairment has prognostic relevance across all disease stages. Collectively, these findings suggest that the prognostic impact of body composition may be stage dependent and tends to become more apparent as the disease progresses.

This study has important clinical implications. According to current clinical guidelines, patients undergoing treatment surveillance are routinely required to undergo contrast-enhanced CT scans of the chest, abdomen, and pelvis every 6–12 weeks [41], which provides an objective basis for standardized, non-invasive quantitative assessment at the third lumbar vertebral level and enables longitudinal monitoring of SMI, SFI, and VFI. Beyond the prognostic value of individual parameters, a composite index derived from these measures may more effectively identify patients at risk of concurrent skeletal muscle and adipose tissue depletion. Recent evidence suggests that reductions in skeletal muscle mass are associated with attenuated responses to immune checkpoint inhibitors [42, 43], while the distribution and quality of adipose tissue may also influence the tumor immune microenvironment [31]. From a clinical perspective, declines in SMI may be improved through individualized nutritional support and structured exercise interventions [44], whereas adipose tissue abnormalities may require integrated management incorporating metabolic regulation and immunonutritional strategies [45, 46]. The composite index can serve as a practical tool for risk stratification, helping to identify high-risk patients early and guide supportive treatment. Although this study did not analyze immunotherapy prognosis, its potential value in immunotherapy evaluation warrants further exploration.

Notably, unlike prior studies that focused on individual body composition indices [47, 48], this study concurrently evaluated SMI, SFI, and VFI to assess their combined prognostic value. Additionally, the retrospective cohort design featured an extended follow-up period of up to 10 years, providing robust long-term outcome data.

It should be noted that 1,294 patients (41% of the initial cohort) were excluded due to missing CT images or having undergone imaging at external hospitals, which may introduce selection bias. However, the included and excluded patients were generally comparable in most key clinical characteristics, including body composition metrics, demographic features, tumor subtypes, and primary treatment modalities, with no significant differences. Although some variables, such as BMI, comorbidities, and pathological stage, showed statistically significant differences, the absolute magnitude of these differences was limited (mostly 5–8%) and were adjusted for as covariates in multivariable analyses (Supplementary Table 1). Therefore, the potential impact of this bias on the internal validity of the study is considered to be manageable.

Nevertheless, several limitations of this study should be acknowledged. As a single-center retrospective analysis, the sample exhibited limited heterogeneity, potentially affecting the generalizability of the findings to the broader population of non-small cell lung cancer patients. In addition, the absence of dynamic data on body composition, as well as information on patients’ physical performance and nutritional interventions, may result in residual confounding. Future large, multicenter prospective studies with longitudinal body composition assessments are needed to validate these findings and clarify the prognostic roles of SMI, SFI, and VFI in patients with NSCLC.

## Conclusion

This study showed that preoperative SMI, SFI, and VFI were associated with survival outcomes in patients with NSCLC. A graded relationship was observed, whereby an increasing number of below-threshold body composition indices was associated with progressively worse OS and RFS. Given the lack of detailed physical fitness data, residual confounding cannot be excluded; nevertheless, the combined assessment of SMI, SFI, and VFI may provide complementary prognostic information and aid risk stratification in patients undergoing surgical resection.

## Supplementary Information

Supplementary Material 1: Supplement Figure 1 Comparison of survival rates based on composite index (number of low-value indices: skeletal muscle index, subcutaneous fat index, and visceral fat index) across pathological stages. 3-Year Survival (A); 5-Year Survival (B).

Supplementary Material 2.

Supplementary Material 3.

Supplementary Material 4.

Supplementary Material 5.

## Data Availability

We sincerely regret that the data used in this study cannot be made publicly available. The datasets involve sensitive patient information, and due to the retrospective nature of the study, it was not possible to obtain informed consent from all participants. Moreover, our research is subject to institutional confidentiality agreements, which limit the ability to share these data externally. We kindly ask for your understanding and are happy to provide further methodological details upon request. Regarding the original data of this study, please contact the corresponding author, Dingyun You, via email at youdingyun@kmmu.edu.cn.

## Abbreviations

BMI: Body mass index
CEA: Carcinoembryonic Antigen
CI: Confidence interval
CT: Computed tomography
HR: Hazard ratio
HU: Hounsfield unit
IQR: Interquartile range
L3: Third lumbar vertebra
NSCLC: Non-small cell lung cancer
OS: Overall survival
RFS: Relapse-free survival
SAT: Subcutaneous adipose tissue
SFI: Subcutaneous fat index
SMA: Skeletal muscle area
SMI: Skeletal muscle index
STROBE: Strengthening the Reporting of Observational Studies in Epidemiology
VAT: Visceral adipose tissue
VFI: Visceral fat index
