# Supplementary figures and images for: Impact of combined skeletal muscle index, subcutaneous fat index, and visceral fat index on prognosis in non-metastatic non-small cell lung cancer
Source: BMC Pulm Med. 2026 Mar 12;26:198. doi: 10.1186/s12890-026-04235-w (PMC13126992; doi:10.1186/s12890-026-04235-w)

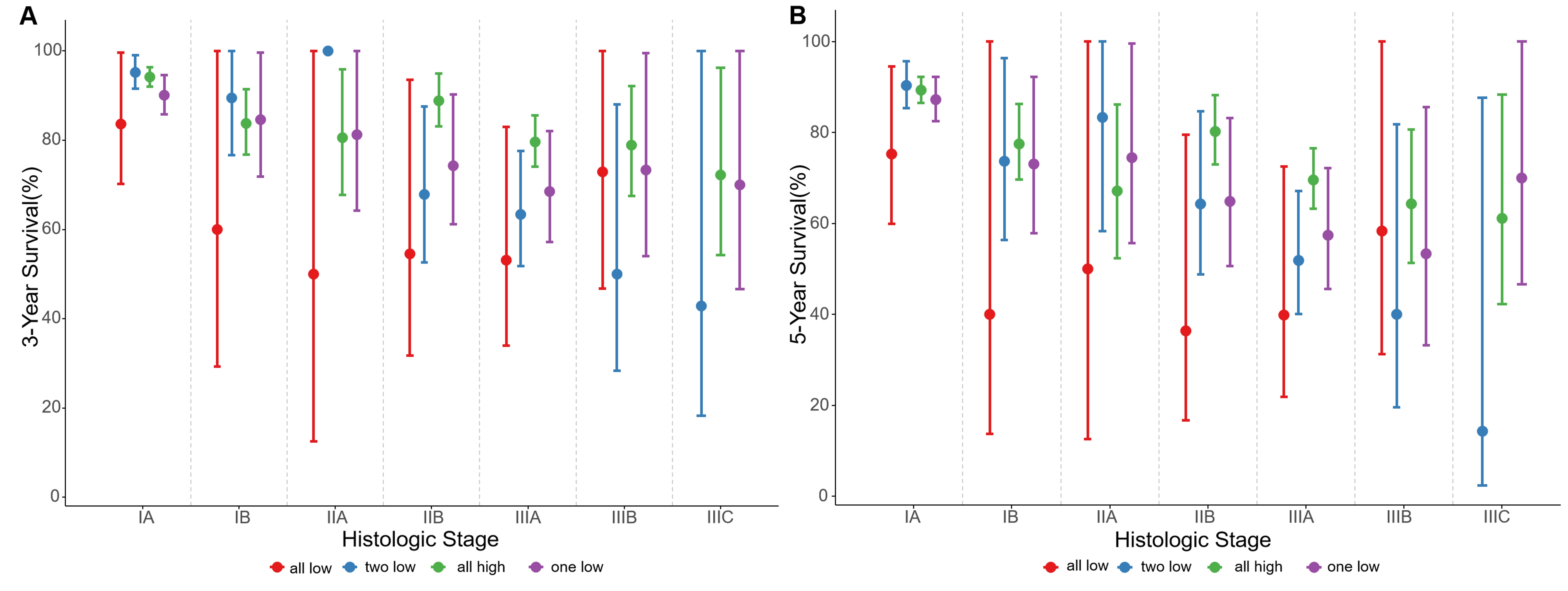

Supplement: Supplementary file 1 — Supplementary Material 1: Supplement Figure 1 Comparison of survival rates based on composite index (number of low-value indices: skeletal muscle index, subcutaneous fat index, and visceral fat index) across pathological stages. 3-Year Survival (A); 5-Year Survival (B). [file 12890_2026_4235_MOESM1_ESM.jpg]
