# Supplementary material for: Impact of combined skeletal muscle index, subcutaneous fat index, and visceral fat index on prognosis in non-metastatic non-small cell lung cancer
Source: BMC Pulm Med. 2026 Mar 12;26:198. doi: 10.1186/s12890-026-04235-w (PMC13126992; doi:10.1186/s12890-026-04235-w)
Supplement: Supplementary file 2 — Supplementary Material 2. [file 12890_2026_4235_MOESM2_ESM.docx]

**Supplement Table 1** Baseline characteristics of eligible versus excluded patients

| **Variable^a^** | **Total^b^**  **(N = 3125)** | **Inclusion^b^**  **(N = 1661)** | **Exclusion^b^**  **(N = 1464)** | ***p*^c^** |
| --- | --- | --- | --- | --- |
| SMI Median (IQR) | 43.9 (38.1, 49.9) | 43.9 (38.2, 50.0) | 43.3 (37.4, 48.5) | 0.202 |
| SAI Median (IQR) | 39.5 (25.6, 58.0) | 39.3 (25.6, 57.2) | 45.9 (23.7, 64.1) | 0.394 |
| VAI Median (IQR) | 31.0 (15.6, 49.7) | 30.7 (15.6, 49.4) | 34.8 (17.0, 51.3) | 0.443 |
| Age,years Mean(SD) | 58.9 (9.6) | 58.8 (9.5) | 58.9 (9.8) | 0.643 |
| Sex |  |  |  | 0.302 |
| Male | 1686 (54.0) | 911 (54.8) | 775 (52.9) |  |
| Female | 1439 (46.0) | 750 (45.2) | 689 (47.1) |  |
| BMI, kg/m^2^ |  |  |  | <0.001 |
| < 18.5 | 159 ( 5.1) | 86 ( 5.2) | 73 ( 5.0) |  |
| 18.5-24.9 | 2315 (74.1) | 1264 (76.1) | 1051 (71.8) |  |
| ≥25 | 641 (20.5) | 311 (18.7) | 330 (22.5) |  |
| Unknown | 10 ( 0.3) | 0 ( 0.0) | 10 ( 0.7) |  |
| Smoking |  |  |  | 0.071 |
| Yes | 1255 (40.2) | 672 (40.5) | 583 (39.8) |  |
| No | 1819 (58.2) | 970 (58.4) | 849 (58.0) |  |
| Unknown | 51 ( 1.6) | 19 ( 1.1) | 32 ( 2.2) |  |
| Hypertension |  |  |  | <0.001 |
| Yes | 430 (13.8) | 226 (13.6) | 204 (13.9) |  |
| No | 2120 (67.8) | 1178 (70.9) | 942 (64.3) |  |
| Unknown | 575 (18.4) | 257 (15.5) | 318 (21.7) |  |
| Diabetes mellitus |  |  |  | <0.001 |
| Yes | 150 ( 4.8) | 81 ( 4.9) | 69 ( 4.7) |  |
| No | 2397 (76.7) | 1323 (79.7) | 1074 (73.4) |  |
| Unknown | 578 (18.5) | 257 (15.5) | 321 (21.9) |  |
| COPD |  |  |  | <0.001 |
| Yes | 99 ( 3.2) | 57 ( 3.4) | 42 ( 2.9) |  |
| No | 2448 (78.3) | 1346 (81.0) | 1102 (75.3) |  |
| Unknown | 578 (18.5) | 258 (15.5) | 320 (21.9) |  |
| Histologic type |  |  |  | 0.417 |
| Adenocarcinoma | 2412 (77.2) | 1283 (77.2) | 1129 (77.1) |  |
| Squamous cell carcinoma | 552 (17.7) | 300 (18.1) | 252 (17.2) |  |
| Other | 161 ( 5.2) | 78 ( 4.7) | 83 ( 5.7) |  |
| Pathological stage |  |  |  | <0.001 |
| Ⅰ | 1745 (55.8) | 959 (57.7) | 786 (53.7) |  |
| Ⅱ | 439 (14.0) | 249 (15.0) | 190 (13.0) |  |
| Ⅲ | 810 (25.9) | 453 (27.3) | 357 (24.4) |  |
| Unknown | 131 ( 4.2) | 0 ( 0.0) | 131 ( 8.9) |  |
| N stage |  |  |  | 0.448 |
| <N2 | 2452 (78.5) | 1310 (78.9) | 1142 (78.0) |  |
| ≥N2 | 559 (17.9) | 297 (17.9) | 262 (17.9) |  |
| Unknown | 114 ( 3.6) | 54 ( 3.3) | 60 ( 4.1) |  |
| Tumor location |  |  |  | 0.719 |
| Upper lobe | 1570 (50.2) | 840 (50.6) | 730 (49.9) |  |
| Non–upper lobe | 1555 (49.8) | 821 (49.4) | 734 (50.1) |  |
| Preoperative CEA,ng/mL |  |  |  | <0.001 |
| <5 | 2023 (64.7) | 1112 (66.9) | 911 (62.2) |  |
| ≥5 | 839 (26.8) | 440 (26.5) | 399 (27.3) |  |
| Unknown | 263 ( 8.4) | 109 ( 6.6) | 154 (10.5) |  |
| Chemotherapy |  |  |  | 0.543 |
| Yes | 1522 (48.7) | 800 (48.2) | 722 (49.3) |  |
| No | 1603 (51.3) | 861 (51.8) | 742 (50.7) |  |
| Radiotherapy |  |  |  | 0.09 |
| Yes | 209 ( 6.7) | 107 ( 6.4) | 102 ( 7.0) |  |
| No | 2882 (92.2) | 1542 (92.8) | 1340 (91.5) |  |
| Unknown | 34 ( 1.1) | 12 ( 0.7) | 22 ( 1.5) |  |

**Note:**

**^a^**Abbreviations: BMI, Body mass index; CEA, **Carcinoembryonic Antigen; IQR, Interquartile range**; COPD, Chronic obstructive pulmonary disease**.**

**^b^**Data are median (IQR)/ Mean (SD) or n (%).

**^c^***p*, using Wilcoxon Mann-Whitney test, chi-square test or exact Fisher test depending on whether the variable is continuous or categorical.
