# Supplementary material for: Impact of combined skeletal muscle index, subcutaneous fat index, and visceral fat index on prognosis in non-metastatic non-small cell lung cancer
Source: BMC Pulm Med. 2026 Mar 12;26:198. doi: 10.1186/s12890-026-04235-w (PMC13126992; doi:10.1186/s12890-026-04235-w)
Supplement: Supplementary file 3 — Supplementary Material 3. [file 12890_2026_4235_MOESM3_ESM.docx]

# **Supplement Table 2** Sensitivity analysis of the association between body composition and overall survival

| **Variable**^a^ | **N (%)** | **Univariate analysis** | |  | **Multivariate analysis**^b^ | |
| --- | --- | --- | --- | --- | --- | --- |
|  |  | **HR (95% CI)**^a^ | ***p*** |  | **HR (95% CI)**^a^ | ***p*** |
| SMI |  |  |  |  |  |  |
| High | 1333 (83.6) | Ref |  |  | Ref |  |
| Low | 262 (16.4) | 1.60 (1.27-2.01) | <0.001 |  | 1.45 (1.12-1.88) | 0.005 |
| SFI |  |  |  |  |  |  |
| High | 1125 (70.5) | Ref |  |  | Ref |  |
| Low | 470 (29.5) | 1.32 (1.08-1.61) | 0.008 |  | 1.59 (1.26-2.02) | <0.001 |
| VFI |  |  |  |  |  |  |
| High | 1287 (80.7) | Ref |  |  | Ref |  |
| Low | 308 (19.3) | 1.55 (1.24-1.93) | <0.001 |  | 1.69 (1.31-2.18) | <0.001 |
| Composite index* |  |  |  |  |  |  |
| All high | 938 (58.8) | Ref |  |  | Ref |  |
| One low | 338 (21.2) | 1.29 (1.02-1.65) | 0.036 |  | 1.53 (1.18-1.99) | 0.001 |
| Two low | 255 (16.0) | 1.39 (1.07-1.81) | 0.014 |  | 1.75 (1.31-2.34) | <0.001 |
| All low | 64 (4.0) | 2.68 (1.84-3.91) | <0.001 |  | 2.88 (1.80-4.63) | <0.001 |

**Note:**

^a^Abbreviations：CI, confidence interval; HR, hazard ratio; Ref, reference; SFI, Subcutaneous fat index; SMI, Skeletal muscle index; VFI, Visceral fat index.

^b^Multivariate analysis was adjusted for Sex, Age, Smoking history, Hypertension, Diabetes mellitus, Chronic obstructive pulmonary disease, BMI, CEA, N stage, Chemotherapy, Radiotherapy, Pathological stage, Tumor location, Histologic type.

*Composite index, number of low values in SMI, SFI, and VFI.
