# Supplementary material for: Impact of combined skeletal muscle index, subcutaneous fat index, and visceral fat index on prognosis in non-metastatic non-small cell lung cancer
Source: BMC Pulm Med. 2026 Mar 12;26:198. doi: 10.1186/s12890-026-04235-w (PMC13126992; doi:10.1186/s12890-026-04235-w)
Supplement: Supplementary file 4 — Supplementary Material 4. [file 12890_2026_4235_MOESM4_ESM.docx]

# **Supplement Table 3** Multivariable-adjusted analyses of skeletal muscle index, subcutaneous fat index, visceral fat index, and composite index in relation to overall survival and relapse-free survival across pathological stage

| **Variable**^a^ | **N (%)** | **Overall Survival** | |  | **Relapse-free Survival** | |
| --- | --- | --- | --- | --- | --- | --- |
|  |  | **HR (95% CI)**^a^ | ***p***^b^ |  | **HR (95% CI)**^a^ | ***p***^b^ |
| ***Stage I and II*** |  |  |  |  |  |  |
| SMI |  |  |  |  |  |  |
| High | 1009(83.5) | Ref |  |  | Ref |  |
| Low | 199 (16.5) | 1.50 (1.07-2.08) | 0.017 |  | 1.39 (1.05-1.84) | 0.022 |
| SFI |  |  |  |  |  |  |
| High | 856 (70.9) | Ref |  |  | Ref |  |
| Low | 352 (29.1) | 1.33 (0.97-1.81) | 0.073 |  | 1.27 (0.97-1.65) | 0.081 |
| VFI |  |  |  |  |  |  |
| High | 989 (81.9) | Ref |  |  | Ref |  |
| Low | 219 (18.1) | 1.67 (1.18-2.37) | 0.004 |  | 1.17 (0.86-1.58) | 0.322 |
| Composite index* |  |  |  |  |  |  |
| All high | 712 (58.9) | Ref |  |  | Ref |  |
| One low | 265 (21.9) | 1.44 (1.02-2.03) | 0.036 |  | 1.29 (0.96-1.72) | 0.088 |
| Two low | 188 (15.6) | 1.24 (0.81-1.87) | 0.320 |  | 1.09 (0.77-1.54) | 0.644 |
| All low | 43 (3.6) | 3.63 (2.06-6.38) | <0.001 |  | 2.59 (1.56-4.32) | <0.001 |
| ***Stage III*** |  |  |  |  |  |  |
| SMI |  |  |  |  |  |  |
| High | 375 (82.8) | Ref |  |  | Ref |  |
| Low | 78 (17.2) | 1.66 (1.11-2.49) | 0.015 |  | 1.43 (1.01-2.03) | 0.042 |
| SFI |  |  |  |  |  |  |
| High | 320 (70.6) | Ref |  |  | Ref |  |
| Low | 133 (29.4) | 1.65 (1.15-2.37) | 0.006 |  | 1.43 (1.03-1.98) | 0.032 |
| VFI |  |  |  |  |  |  |
| High | 349 (77.0) | Ref |  |  | Ref |  |
| Low | 104 (23.0) | 1.72 (1.18-2.51) | 0.005 |  | 1.38 (0.98-1.94) | 0.065 |
| Composite index* |  |  |  |  |  |  |
| All high | 267 (58.9) | Ref |  |  | Ref |  |
| One low | 83 (18.3) | 1.68 (1.11-2.53) | 0.013 |  | 1.70 (1.18-2.43) | 0.004 |
| Two low | 77 (17.0) | 2.47 (1.63-3.75) | <0.001 |  | 1.74 (1.19-2.55) | 0.005 |
| All low | 26 (5.7) | 1.61 (0.73-3.53) | 0.237 |  | 1.54 (0.81-2.92) | 0.190 |

**Note:**

^a^Abbreviations：CI, confidence interval; HR, hazard ratio; Ref, reference; SFI, Subcutaneous fat index; SMI, Skeletal muscle index; VFI, Visceral fat index.

^b^Multivariate analysis was adjusted for Sex, Age, Smoking history, Hypertension, Diabetes mellitus, Chronic obstructive pulmonary disease, BMI, CEA, N stage, Chemotherapy, Radiotherapy, Tumor location, Histologic type.

*Composite index, number of low values in SMI, SFI, and VFI.
