# Supplementary material for: Impact of combined skeletal muscle index, subcutaneous fat index, and visceral fat index on prognosis in non-metastatic non-small cell lung cancer
Source: BMC Pulm Med. 2026 Mar 12;26:198. doi: 10.1186/s12890-026-04235-w (PMC13126992; doi:10.1186/s12890-026-04235-w)
Supplement: Supplementary file 5 — Supplementary Material 5. [file 12890_2026_4235_MOESM5_ESM.pdf]

Review Article

# Recurrence after surgery in patients with NSCLC

**Hidetaka Uramoto, Fumihiro Tanaka**

Second Department of Surgery, University of Occupational and Environmental Health, Kitakyushu, Japan

*Correspondence to:* Hidetaka Uramoto. Second Department of Surgery, School of Medicine, University of Occupational and Environmental Health, 1-1 Iseigaoka, Yahatanishi-ku, Kitakyushu 807-8555, Japan. Email: hidetaka@med.uoeh-u.ac.jp.

**Abstract:** Surgery remains the only potentially curative modality for early-stage non-small cell lung cancer (NSCLC) patients and tissue availability is made possible. However, a proportion of lung cancer patients develop recurrence, even after curative resection. This review discusses the superiority of surgery, the reasons for recurrence, the timing and pattern of recurrence, the identification of factors related to recurrence, current provisions for treatment and perspectives about surgery for patients with NSCLC.

**Keywords:** Recurrence; relapse; disease-free survival (DFS); surgery; non-small cell lung cancer (NSCLC)

Submitted Nov 15, 2013. Accepted for publication Dec 30, 2013.
doi: 10.3978/j.issn.2218-6751.2013.12.05
**View this article at:** http://dx.doi.org/10.3978/j.issn.2218-6751.2013.12.05

## The superiority of surgery, and its Achilles' heel

Lung cancer has been the most common type of cancer in the world for several decades (1). Surgery remains the only potentially curative treatment for early-stage non-small cell lung cancer (NSCLC) patients, and complete resection can be achieved in a matter of hours and tissue availability is made possible (2). These points are advantages of surgery that are not associated with other modalities, such as chemotherapy and radiotherapy. In fact, many patients with NSCLC have been cured by surgery. However, there are also many cases that fail to achieve a cure following surgery. In fact, 30% to 55% of patients with NSCLC develop recurrence and die of their disease despite curative resection (3-5). Therefore, many patients eventually die of their disease due to recurrence after surgery (6,7). Furthermore, surgery itself possesses a certain amount of risk (8,9). Although treatment-related death is relatively uncommon, there is still a significant risk of mortality, and most patients experience a decrease in lung function after surgery. Thus, despite the advances in surgery, treatment by surgical resection is less than perfect, even when a complete resection is possible.

## Reasons for recurrence after complete resection

The resection of early stage NSCLC offers patients the best hope of a cure, however, the recurrence rates post-resection remain high. There have been numerous studies conducted to determine why recurrence develops after complete resection for patients with NSCLC. These studies have shown that, right from the start, complete removal needs to be ensured both macroscopically and microscopically. It has been suggested that there are often occult micro-metastatic cancer cells, which are undetected by standard staging methods, such as modern diagnostic imaging, already present systemically at the time of surgery, suggesting that there is an underestimation of the true tumor stage. Second, handling of the tumor during surgery itself might lead to the dissemination of cancer cells (10,11). Based on former speculation, reports regarding disseminated tumor cells (DTC) or circulating tumor cells (CTC) have been described (12-14). A number of studies have reported an association between the presence of DTC or CTC during tumor resection and the patient outcome (12). However, some controversy exists regarding this theory (15). Therefore, the detection of DTC or CTC is currently not included in the NSCLC staging or treatment recommendations, and it is also currently unclear whether these cells have proliferative activity or are so-called "dormant cells" (16).

## The timing and pattern of recurrence

Conventionally, recurrence most commonly occurs at

© Translational lung cancer research. All rights reserved.

distant sites (6,17). On the other hand, there was a report concerning the underestimation of the frequency of local recurrence. In that study, Boyd *et al*. examined the timing of local and distant failure of 250 patients who developed recurrent among 975 patients who underwent surgery (18). Seventeen percent, 44% and 39% of the recurrences were confined to local, distant and both sites, respectively. Only 0.6% of the patients developed a sequential local recurrence, suggesting that the crude rate of local failure might be underestimated if only the first sites of failure are reported. Distant metastases and local recurrence might arise from occult cancer cells or due to dissemination during surgery, respectively, as described above.

## The identification of factors related to recurrence following surgery

The TNM staging indicates the level of disease progression and the malignant potential of the primary lung cancer (19). However, even patients with disease at the same stage exhibit wide variations in their incidence of recurrence after curative resection. Therefore, the current TNM staging system, which is based on clinical and pathological findings, may have reached the limit of its usefulness (20). Accurately predicting the cases in which disease is likely to recur can help guide the administration of adjuvant therapies, not only to those most likely to benefit from them (21,22), but also to those eligible for complete resection, because surgery itself possesses a certain amount of risk (8,9). There are two methods for identifying factors related to recurrence following surgery (*Table 1*).

One is classical judgment using clinical parameters, in addition to the TNM classification. Tumor markers, such as CEA, were proven to be independent factors predicting the risk of recurrence (23). Furthermore, the physical examination is very important, because a lower performance status (PS) and the presence of symptoms are unfavorable prognostic factors for the disease free survival (DFS) (24). The standard uptake value (SUV) in PET has also been reported to be a significant independent factor predicting the DFS (25). An extensive pathological investigation is also important, because the histological differentiation, vessel invasion, lymphatic permeation and pleural invasion have all been reported to be poor prognostic factors for the DFS (26-28). Furthermore, complete mediastinal lymph node (MLN) dissection is associated with improved survival in comparison to random lymph node sampling for the patients with stage I NSCLC (29), although there have been some negative reports (30).

The other method for predicting recurrence uses molecular biological techniques. Lung cancer is a highly aggressive neoplastic disease that includes different histological subtypes with distinct clinicopathological and molecular features (6,7). Therefore, the establishment of useful markers is necessary to accurately classify early- and advanced-stage disease. The combined use of the KRAS status and the Ki-67 expression level was an excellent prognostic marker to predict the postoperative recurrence of stage I adenocarcinoma (31). The clinical usefulness of Ki-67, MACC, TS or IGF1R, but not PCNA or the EMT status, in primary lung adenocarcinoma has been reported (17,32-36). Poleri *et al*. showed that MIB-1 and Bcl-2 are independent prognostic factors for recurrence in stage IB patients (37). Brock *et al*. reported that methylation of the promoter regions of p16 and CDH13 in both the tumor and MLNs are associated with recurrence for patients with stage I NSCLC (38). An assessment of the combination of the several protein expression indicated that the cyclin E-negative/p27-positive group or higher expression of CXCR7 had a significantly higher DFS rate than did other groups (39,40). Many other markers have been reported (41-44). On the other hand, there have been some negative findings (45-48). Pastorino *et al*. reported that none of the immunocytochemical markers such as laminin receptor; EGFR, c-erbB2/Neu, BCl2, and p53, emerged as an independent predictive factor for survival (48).

Several attempts have been made to detect micrometastatic tumor cells or DTC in lymph nodes (LNs), bone marrow, and CTC in the peripheral blood (49,50). The DFS curves demonstrated that patients with cytokeratin (CK) cells in the pN0 LNs had significantly shorter survival periods than those without CK cells (51). CK19 mRNA in MLNs is significantly associated with an increased risk of rapid recurrence (52), which is consistent with our recent data (6). However, in contrast to the previously published data, Poncelet *et al*. described that the presence of occult micrometastasis had no influence on the DFS (53). A study by Hsu *et al*. also showed that the occurrence of bone marrow microinvolvement is not a good predictor of the long-term prognosis (54).

The development of the microarray analyses has made it possible to simultaneously measure the expression levels of thousands of genes (20,55-57). Tumor-specific genetic fingerprints/gene signatures can affect the prognosis (58). The deregulation of microRNAs is also linked to cancer initiation and progression, indicating that microRNAs may act as tumor suppressor genes or oncogenes (20). In fact, the

© Translational lung cancer research. All rights reserved.

**Table 1** Parameters predicting the recurrence after complete resection for patients with NSCLC in the literature

| Parameters | Significance |
|---|---|
| Clinical parameters | |
| High CEA, lymphatic permeation, and pleural invasion | High CEA level, lymphatic permeation, pleural invasion and, perioperative transfusion were proven to be independent factors for overall recurrence |
| Histological differentiation, vessel invasion, and visceral pleural invasion | Histological differentiation, vessel invasion, and visceral pleural invasion in stage I and AD histology and visceral pleural invasion in stage IIN0 and stage IIN1 were shown to be independent significant risk factors for recurrence |
| Intratumoral vascular invasion and nodal involvement | Intratumoral vascular invasion and nodal involvement significantly influenced recurrence five years after complete resection |
| Intratumoral blood vessel invasion | Independent prognostic factor in poor DF |
| SUV | Patients with high max SUV and LVI were more likely to have recurrence |
| Number of LNs | Systematic sampling and complete MLNs dissection were associated with improved survival in comparison to random LNs sampling |
| PS, and symptoms at recurrence, liver recurrence, stage IIB or worse, and multiple recurrences | Strongly associated with post-recurrence survival |
| Molecular parameters | |
| KRAS and Ki-67 | Excellent prognostic marker to predict the postoperative recurrence of stage I AD |
| Ki-67 | Ki-67 expression was independently associated with an increased risk of poor DFS |
| MIB-1 and Bcl-2 | The mitosis count and MIB-1 expression significantly correlated with recurrence and Bcl-2 tumors had a poor outcome |
| p16 and CDH1 | Methylation of the promoter regions of p16 and CDH13 in both tumor and MLNs were associated with recurrence for patients with stage I NSCLC |
| MACC | MACC1 gene amplification may be a useful marker for predicting postoperative recurrence |
| MACC | Positive staining for MACC1 expression in resected specimens was associated with a poorer DFS |
| CXCR7 | A higher expression of CXCR7 is associated with poor DFS in patients with p-stage I NSCLC |
| TS | A strong TS expression may be a useful marker for predicting postoperative recurrence in patients with lung AD following surgery |
| CK19 | CK19 mRNA detected by RT-PCR in MLNS was significantly associated with an increased risk of rapid recurrence |
| CK | The DFS curves demonstrated that the patients with CK cells in the pN0 LNs had significantly shorter survival periods than those without CK cells |
| microRNA expression profiles | MicroRNA expression profiles predicted recurrence of stage I NSCLC after surgical resection |
| EGFR mutations | Activating mutations within the EGFR TK domain can be used to predict the risk of recurrence in curatively resected pulmonary AD |
| IGF1R | Positive staining for IGF1R expression was an independent factor associated with tumor recurrence |
| EMT | EMT does not provide any relevant prognostic information about lung adenocarcinoma |
| Micro RNA | The micro RNA expression in resected NSCLC could potentially identify those at high risk of relapse after surgery |

AD, adenocarcinoma; LN, lymph node; PS, performance status; MLNs, mediastinal lymph nodes; SUV, standard uptake value; miRNAs, microRNAs.

© Translational lung cancer research. All rights reserved.

microRNA expression in resected NSCLC could potentially identify those at high risk of relapse after surgery (59). Despite these findings, it is still a challenge to assign a prognostic value to such molecular pathways because of the poor reproducibility of findings (60). The biomarker might have more clinical relevance if its impact on the response to chemotherapy could be predicted at the same time. Despite a great deal of an effort to identify of factors related to recurrence following surgery, there is currently no consensus concerning the use of biomarkers to predict NSCLC recurrence. Therefore, clinical trials to investigate predictive factors with recurrence as one of the endpoints are needed.

## Current provisions

At this time, there are two countermeasures to prevent recurrence; preoperative induction and postoperative adjuvant chemotherapy. Induction therapy possesses some advantages (61), because (I) patients receive the full dose of the planned regimen and have good compliance because of the preoperative setting. In other words, a considerable number of patients are unable to receive adjuvant chemotherapy due to slow recovery from surgery. In fact, in a previous study, a greater proportion (90%) of patients in the neoadjuvant group received the planned chemotherapy compared with the adjuvant group, in which only 66% of the patients started adjuvant treatment (62); (II) The response to the treatment modality remains unaffected by surgery, such as a decline in lung function; (III) The treatment can obliterate occult microscopic systemic disease, and is possible to test the sensitivity *in vivo*, which can be useful to select the regimen used to treat any recurrence; (IV) Chemotherapy can cause the down-staging of the local lesion, and increase the complete resection rate (61,63). Therefore, neoadjuvant chemotherapy is an attractive treatment option. However, the potential disadvantages of neoadjuvant chemotherapy are (I) treatment-related toxicities can lead to a delay in surgery; (II) the changes after chemotherapy might lead to difficulties with the surgery; (III) neoadjuvant treatment can increase the perioperative complications; (IV) treatment can lead to a missed opportunity to perform surgery by exacerbating the disease (61). So far, the prospective trials of neoadjuvant chemotherapy for NSCLC were all negative with regard to survival (62,64-67). Therefore, neoadjuvant chemotherapy is still considered an experimental treatment modality for patients.

Another strategy is the addition of thoracic radiotherapy to chemotherapy in the preoperative setting, which may improve the local control and help sterilize mediastinal disease. However, the North American Intergroup Trial group and German Lung Cancer Cooperative Group, and a Swiss group, did not find superior survival in the patients assigned to the thoracic radiotherapy plus chemotherapy group (68-70). Therefore, there is currently no clear evidence favoring a neoadjuvant strategy compared with adjuvant strategies for early-stage NSCLC (61).

On the other hand, there is lot of evidence supporting postoperative adjuvant chemotherapy. The Lung Adjuvant Cisplatin Evaluation (LACE) meta-analysis clearly demonstrated a trend toward a benefit in stage IB NSCLC and clear benefit for patients with stage II N1 and IIIA (mostly N2 cases) patients, with an average benefit of 5% at five years (71). Consequently, adjuvant chemotherapy is recommended for patients with resected stage II-III NSCLC (72). However, chemotherapy compliance in the post-surgery setting is relatively poor (22). Nevertheless, the combined hazard ratios (HR) from this meta-analysis suggested that the estimated benefit from preoperative chemotherapy was similar in magnitude to that expected with postoperative chemotherapy, in spite of the large difference in compliance (62,73).

For patients with early-stage NSCLC, surgery remains the treatment of choice, as described above. At present, stereotactic body radiation therapy (SBRT) has been shown to provide a benefit for patients who are not fit for surgery or those who refuse surgery (74). However, the quality of the instruments used to deliver radiation therapy has improved dramatically. SBRT has emerged as a new treatment modality to completely eradicate the primary tumor. Excellent outcomes from SBRT, including good long-term survival, and favorable comparisons to groups of patients treated with standard surgery have been reported (75). However, these reports were retrospective. The head-to-head comparison of SBRT versus surgery for operable patients seems to be very difficult to conduct from the standpoint of the recruiting, decisions regarding the endpoints, stage migration and tissue availability. In fact, such trials have already closed due to a lack of accrual (76,77). This subject will need to be examined in the future. On the other hand, for locally-advanced disease, the impact of neoadjuvant therapy followed by surgery has failed to show a survival benefit (78-81). Therefore, surgical resection is the best treatment option at present. However, patients with clinical stage IIIA-N2 NSCLC who underwent neoadjuvant chemoradiotherapy followed

© Translational lung cancer research. All rights reserved.

by lobectomy, were found to have an improved survival based on the data from the American College of Surgeons Commission on Cancer (82). Moreover, a histopathological assessment of resected specimens after neoadjuvant chemotherapy could potentially have a role, in addition to evaluating the response to chemotherapy (83). This patient selection is important for induction treatment before surgery to improve the outcome. Recent developments in molecularly-targeted drugs have transformed cancer therapy in remarkable ways. A multicenter phase II randomized study of customized neoadjuvant therapy versus standard chemotherapy has been open for accrual. Patients will be randomized to receive either standard chemotherapy or customized therapy before resection using predetermined values for *ERCC1*, *RRM1*, *TS* and *EGFR* mutations (84). The West Japan Oncology Group has already launched a randomized phase III trial comparing adjuvant gefitinib with chemotherapy in patients with completely resected stage II-III NSCLC harboring *EGFR* mutations (85). Further studies with targeted therapy before and after surgery are warranted.

## Current perspective

The standard treatment for early-stage NSCLC patients is still surgery. Surgery is also of use, because it provides tissue specimens to evaluate, so that an appropriate treatment can be defined based on the accurate evaluation of the tumor, biologically. A better individualization of treatment approaches requires a more precise understanding of the molecular features of lung cancer (5). However, there are some real limitations to surgery, as pointed out above. Additional studies are needed to reduce the incidence of recurrence after surgery and to perform more accurate patient selection to ensure a radical cure.

## Acknowledgements

This study was supported, in part, by a Grant-in-Aid for Scientific Research (C) 25462202 from the Ministry of Education, Culture, Sports, Science and Technology (MEXT), Japan and a UOEH Research Grant for Promotion of Occupational Health
*Disclosure:* The authors declare no conflict of interest.

## References

1. Jemal A, Bray F, Center MM, et al. Global cancer statistics. CA Cancer J Clin 2011;61:69-90.
2. Mitsudomi T, Suda K, Yatabe Y. Surgery for NSCLC in the era of personalized medicine. Nat Rev Clin Oncol 2013;10:235-44.
3. al-Kattan K, Sepsas E, Fountain SW, et al. Disease recurrence after resection for stage I lung cancer. Eur J Cardiothorac Surg 1997;12:380-4.
4. Hoffman PC, Mauer AM, Vokes EE. Lung cancer. Lancet 2000;355:479-85.
5. Carnio S, Novello S, Papotti M, et al. Prognostic and predictive biomarkers in early stage non small-cell lung cancer: tumor based approaches including gene signatures. Transl Lung Cancer Res 2013;2:372-81.
6. Yamashita T, Uramoto H, Onitsuka T, et al. Association between lymphangiogenesis-/micrometastasis- and adhesion-related molecules in resected stage I NSCLC. Lung Cancer 2010;70:320-8.
7. Uramoto H, Tanaka F. Prediction of recurrence after complete resection in patients with NSCLC. Anticancer Res 2012;32:3953-60.
8. Uramoto H, Nakanishi R, Fujino Y, et al. Prediction of pulmonary complications after a lobectomy in patients with non-small cell lung cancer. Thorax 2001;56:59-61.
9. Uramoto H, Takenoyama M, Hanagiri T. Simple prophylactic fixation for lung torsion. Ann Thorac Surg 2010;90:2028-30.
10. Hayashi N, Egami H, Kai M, et al. No-touch isolation technique reduces intraoperative shedding of tumor cells into the portal vein during resection of colorectal cancer. Surgery 1999;125:369-74.
11. Hashimoto M, Tanaka F, Yoneda K, et al. Significant increase in circulating tumour cells in pulmonary venous blood during surgical manipulation in patients with primary lung cancer. 27$^{th}$ European Association for Cardio-Thoracic Surgery (EACTS), 2013, Vienna, Austria (abstract #090).
12. Pantel K, Brakenhoff RH, Brandt B. Detection, clinical relevance and specific biological properties of disseminating tumour cells. Nat Rev Cancer 2008;8:329-40.
13. Sienel W, Seen-Hibler R, Mutschler W, et al. Tumour cells in the tumour draining vein of patients with non-small cell lung cancer: detection rate and clinical significance. Eur J Cardiothorac Surg 2003;23:451-6.
14. Tanaka F, Yoneda K, Kondo N, et al. Circulating tumor cell as a diagnostic marker in primary lung cancer. Clin Cancer Res 2009;15:6980-6.
15. Rud AK, Borgen E, Mælandsmo GM, et al. Clinical significance of disseminated tumour cells in non-small cell

© Translational lung cancer research. All rights reserved. 

lung cancer. Br J Cancer 2013;109:1264-70.
16. Bessa X, Elizalde JI, Boix L, et al. Lack of prognostic influence of circulating tumor cells in peripheral blood of patients with colorectal cancer. Gastroenterology 2001;120:1084-92.
17. Nakagawa M, Uramoto H, Oka S, et al. Clinical significance of IGF1R expression in non-small-cell lung cancer. Clin Lung Cancer 2012;13:136-42.
18. Boyd JA, Hubbs JL, Kim DW, et al. Timing of local and distant failure in resected lung cancer: implications for reported rates of local failure. J Thorac Oncol 2010;5:211-4.
19. Brundage MD, Davies D, Mackillop WJ. Prognostic factors in non-small cell lung cancer: a decade of progress. Chest 2002;122:1037-57.
20. Pollack JR. A perspective on DNA microarrays in pathology research and practice. Am J Pathol 2007;171:375-85.
21. Patnaik SK, Kannisto E, Knudsen S, et al. Evaluation of microRNA expression profiles that may predict recurrence of localized stage I non-small cell lung cancer after surgical resection. Cancer Res 2010;70:36-45.
22. Uramoto H, Nakanishi R, Nagashima A, et al. A randomized phase II trial of adjuvant chemotherapy with bi-weekly carboplatin plus paclitaxel versus carboplatin plus gemcitabine in patients with completely resected non-small cell lung cancer. Anticancer Res 2010;30:4695-9.
23. Kawachi R, Tsukada H, Nakazato Y, et al. Early recurrence after surgical resection in patients with pathological stage I non-small cell lung cancer. Thorac Cardiovasc Surg 2009;57:472-5.
24. Pasini F, Pelosi G, Valduga F, et al. Late events and clinical prognostic factors in stage I non small cell lung cancer. Lung Cancer 2002;37:171-7.
25. Shiono S, Abiko M, Sato T. Positron emission tomography/computed tomography and lymphovascular invasion predict recurrence in stage I lung cancers. J Thorac Oncol 2011;6:43-7.
26. Maeda R, Yoshida J, Ishii G, et al. Risk factors for tumor recurrence in patients with early-stage (stage I and II) non-small cell lung cancer: patient selection criteria for adjuvant chemotherapy according to the seventh edition TNM classification. Chest 2011;140:1494-502.
27. Inoue M, Minami M, Sawabata N, et al. Clinical outcome of resected solid-type small-sized c-stage IA non-small cell lung cancer. Eur J Cardiothorac Surg 2010;37:1445-9.
28. Shoji F, Haro A, Yoshida T, et al. Prognostic significance of intratumoral blood vessel invasion in pathologic stage IA non-small cell lung cancer. Ann Thorac Surg 2010;89:864-9.
29. Gajra A, Newman N, Gamble GP, et al. Effect of number of lymph nodes sampled on outcome in patients with stage I non-small-cell lung cancer. J Clin Oncol 2003;21:1029-34.
30. Darling GE, Allen MS, Decker PA, et al. Randomized trial of mediastinal lymph node sampling versus complete lymphadenectomy during pulmonary resection in the patient with N0 or N1 (less than hilar) non-small cell carcinoma: results of the American College of Surgery Oncology Group Z0030 Trial. J Thorac Cardiovasc Surg 2011;141:662-70.
31. Woo T, Okudela K, Yazawa T, et al. Prognostic value of KRAS mutations and Ki-67 expression in stage I lung adenocarcinomas. Lung Cancer 2009;65:355-62.
32. Oka S, Uramoto H, Shimokawa H, et al. The expression of Ki-67, but not proliferating cell nuclear antigen, predicts poor disease free survival in patients with adenocarcinoma of the lung. Anticancer Res 2011;31:4277-82.
33. Chundong G, Uramoto H, Onitsuka T, et al. Molecular diagnosis of MACC1 status in lung adenocarcinoma by immunohistochemical analysis. Anticancer Res 2011;31:1141-5.
34. Shimokawa H, Uramoto H, Onitsuka T, et al. Overexpression of MACC1 mRNA in lung adenocarcinoma is associated with postoperative recurrence. J Thorac Cardiovasc Surg 2011;141:895-8.
35. Shimokawa H, Uramoto H, Onitsuka T, et al. TS expression predicts postoperative recurrence in adenocarcinoma of the lung. Lung Cancer 2011;72:360-4.
36. Chikaishi Y, Uramoto H, Tanaka F. The EMT status in the primary tumor does not predict postoperative recurrence or disease-free survival in lung adenocarcinoma. Anticancer Res 2011;31:4451-6.
37. Poleri C, Morero JL, Nieva B, et al. Risk of recurrence in patients with surgically resected stage I non-small cell lung carcinoma: histopathologic and immunohistochemical analysis. Chest 2003;123:1858-67.
38. Brock MV, Hooker CM, Ota-Machida E, et al. DNA methylation markers and early recurrence in stage I lung cancer. N Engl J Med 2008;358:1118-28.
39. Takahashi S, Kamata Y, Tamo W, et al. Relationship between postoperative recurrence and expression of cyclin E, p27, and Ki-67 in non-small cell lung cancer without lymph node metastases. Int J Clin Oncol 2002;7:349-55.
40. Iwakiri S, Mino N, Takahashi T, et al. Higher expression of chemokine receptor CXCR7 is linked to early and metastatic recurrence in pathological stage I nonsmall cell lung cancer. Cancer 2009;115:2580-93.
41. Steg AD, Bevis KS, Katre AA, et al. Stem cell pathways

© Translational lung cancer research. All rights reserved.

contribute to clinical chemoresistance in ovarian cancer. Clin Cancer Res 2012;18:869-81.
42. Leinonen T, Pirinen R, Böhm J, et al. Increased expression of matrix metalloproteinase-2 (MMP-2) predicts tumour recurrence and unfavourable outcome in non-small cell lung cancer. Histol Histopathol 2008;23:693-700.
43. Ono K, Uramoto H, Hanagiri T. Expression of dysadherin and cytokeratin as prognostic indicators of disease-free survival in patients with stage I NSCLC. Anticancer Res 2010;30:3273-8.
44. Ludovini V, Bellezza G, Pistola L, et al. High coexpression of both insulin-like growth factor receptor-1 (IGFR-1) and epidermal growth factor receptor (EGFR) is associated with shorter disease-free survival in resected non-small-cell lung cancer patients. Ann Oncol 2009;20:842-9.
45. Baksh FK, Dacic S, Finkelstein SD, et al. Widespread molecular alterations present in stage I non-small cell lung carcinoma fail to predict tumor recurrence. Mod Pathol 2003;16:28-34.
46. Lim KH, Huang MJ, Liu HC, et al. Lack of prognostic value of EGFR mutations in primary resected non-small cell lung cancer. Med Oncol 2007;24:388-93.
47. Kosaka T, Yatabe Y, Onozato R, et al. Prognostic implication of EGFR, KRAS, and TP53 gene mutations in a large cohort of Japanese patients with surgically treated lung adenocarcinoma. J Thorac Oncol 2009;4:22-9.
48. Pastorino U, Andreola S, Tagliabue E, et al. Immunocytochemical markers in stage I lung cancer: relevance to prognosis. J Clin Oncol 1997;15:2858-65.
49. Osaki T, Oyama T, Gu CD, et al. Prognostic impact of micrometastatic tumor cells in the lymph nodes and bone marrow of patients with completely resected stage I non-small-cell lung cancer. J Clin Oncol 2002;20:2930-6.
50. Riethdorf S, Wikman H, Pantel K. Review: biological relevance of disseminated tumor cells in cancer patients. Int J Cancer 2008;123:1991-2006.
51. Yasumoto K, Osaki T, Watanabe Y, et al. Prognostic value of cytokeratin-positive cells in the bone marrow and lymph nodes of patients with resected nonsmall cell lung cancer: a multicenter prospective study. Ann Thorac Surg 2003;76:194-201; discussion 202.
52. Le Pimpec-Barthes F, Danel C, Lacave R, et al. Association of CK19 mRNA detection of occult cancer cells in mediastinal lymph nodes in non-small cell lung carcinoma and high risk of early recurrence. Eur J Cancer 2005;41:306-12.
53. Poncelet AJ, Weynand B, Ferdin F, et al. Bone marrow micrometastasis might not be a short-term predictor of survival in early stages non-small cell lung carcinoma. Eur J Cardiothorac Surg 2001;20:481-8.
54. Hsu CP, Shai SE, Hsia JY, et al. Clinical significance of bone marrow microinvolvement in nonsmall cell lung carcinoma. Cancer 2004;100:794-800.
55. Zhu CQ, Pintilie M, John T, et al. Understanding prognostic gene expression signatures in lung cancer. Clin Lung Cancer 2009;10:331-40.
56. Director's Challenge Consortium for the Molecular Classification of Lung Adenocarcinoma, Shedden K, Taylor JM, et al. Gene expression-based survival prediction in lung adenocarcinoma: a multi-site, blinded validation study. Nat Med 2008;14:822-7.
57. Kratz JR, He J, Van Den Eeden SK, et al. A practical molecular assay to predict survival in resected non-squamous, non-small-cell lung cancer: development and international validation studies. Lancet 2012;379:823-32.
58. Santos ES, Blaya M, Raez LE. Gene expression profiling and non-small-cell lung cancer: where are we now? Clin Lung Cancer 2009;10:168-73.
59. Duncavage E, Goodgame B, Sezhiyan A, et al. Use of microRNA expression levels to predict outcomes in resected stage I non-small cell lung cancer. J Thorac Oncol 2010;5:1755-63.
60. Lau SK, Boutros PC, Pintilie M, et al. Three-gene prognostic classifier for early-stage non small-cell lung cancer. J Clin Oncol 2007;25:5562-9.
61. Salvà F, Felip E. Neoadjuvant chemotherapy in early-stage non-small cell lung cancer. Transl Lung Cancer Res 2013;2:398-402.
62. Felip E, Rosell R, Maestre JA, et al. Preoperative chemotherapy plus surgery versus surgery plus adjuvant chemotherapy versus surgery alone in early-stage non-small-cell lung cancer. J Clin Oncol 2010;28:3138-45.
63. Naidoo R, Windsor MN, Goldstraw P. Surgery in 2013 and beyond. J Thorac Dis 2013;5:S593-606.
64. Pisters KM, Ginsberg RJ, Giroux DJ, et al. Induction chemotherapy before surgery for early-stage lung cancer: a novel approach. Bimodality Lung Oncology Team. J Thorac Cardiovasc Surg 2000;119:429-39.
65. Pisters KM, Vallières E, Crowley JJ, et al. Surgery with or without preoperative paclitaxel and carboplatin in early-stage non-small-cell lung cancer: Southwest Oncology Group Trial S9900, an intergroup, randomized, phase III trial. J Clin Oncol 2010;28:1843-9.
66. Gilligan D, Nicolson M, Smith I, et al. Preoperative chemotherapy in patients with resectable non-small cell lung cancer: results of the MRC LU22/NVALT 2/

© Translational lung cancer research. All rights reserved. 

EORTC 08012 multicentre randomised trial and update of systematic review. Lancet 2007;369:1929-37.
67. Scagliotti GV, Pastorino U, Vansteenkiste JF, et al. Randomized phase III study of surgery alone or surgery plus preoperative cisplatin and gemcitabine in stages IB to IIIA non-small-cell lung cancer. J Clin Oncol 2012;30:172-8.
68. Albain KS, Swann RS, Rusch VW, et al. Radiotherapy plus chemotherapy with or without surgical resection for stage III non-small-cell lung cancer: a phase III randomised controlled trial. Lancet 2009;374:379-86.
69. Thomas M, Rübe C, Hoffknecht P, et al. Effect of preoperative chemoradiation in addition to preoperative chemotherapy: a randomised trial in stage III non-small-cell lung cancer. Lancet Oncol 2008;9:636-48.
70. Pless M, Stupp R, Ris HB, et al. Neoadjuvant chemotherapy with or without preoperative irradiation in stage IIIA/N2 non-small cell lung cancer (NSCLC): a randomized phase III trial by the Swiss Group for Clinical Cancer Research (SAKK trial 16/00). J Clin Oncol 2013;31:abstr 7503.
71. Pignon JP, Tribodet H, Scagliotti GV, et al. Lung adjuvant cisplatin evaluation: a pooled analysis by the LACE Collaborative Group. J Clin Oncol 2008;26:3552-9.
72. Vansteenkiste J, De Ruysscher D, Eberhardt WE, et al. Early and locally advanced non-small-cell lung cancer (NSCLC): ESMO Clinical Practice Guidelines for diagnosis, treatment and follow-up. Ann Oncol 2013;24 Suppl 6:vi89-98.
73. Scagliotti GV, Pastorino U, Vansteenkiste JF, et al. Randomized phase III study of surgery alone or surgery plus preoperative cisplatin and gemcitabine in stages IB to IIIA non-small-cell lung cancer. J Clin Oncol 2012;30:172-8.
74. Nagata Y, Hiraoka M, Shibata T, et al. Stereotactic body radiation therapy for T1N0M0 non-small cell lung cancer: first report for inoperable population of a Phase II Trial by Japan Clinical Oncology Group (JCOG 0403). Int J Radiat Oncol Biol Phys 2012;83:S46 .
75. Onishi H, Shirato H, Nagata Y, et al. Hypofractionated stereotactic radiotherapy (HypoFXSRT) for stage I non-small cell lung cancer: updated results of 257 patients in a Japanese multi-institutional study. J Thorac Oncol 2007;2:S94-100.
76. Hurkmans CW, Cuijpers JP, Lagerwaard FJ, et al. Recommendations for implementing stereotactic radiotherapy in peripheral stage IA non-small cell lung cancer: report from the Quality Assurance Working Party of the randomised phase III ROSEL study. Radiat Oncol 2009;4:1.
77. Available online: http://clinicaltrials.gov/ct2/show/NCT00840749
78. Nagai K, Tsuchiya R, Mori T, et al. A randomized trial comparing induction chemotherapy followed by surgery with surgery alone for patients with stage IIIA N2 non-small cell lung cancer (JCOG 9209). J Thorac Cardiovasc Surg 2003;125:254-60.
79. Albain KS, Swann RS, Rusch VR, et al. Phase III study of concurrent chemotherapy and radiotherapy (CT/RT) vs CT/RT followed by surgical resection for stage IIIA(pN2) non-small cell lung cancer (NSCLC): outcomes update of North American Intergroup 0139 (RTOG 9309). J Clin Oncol 2005:23;abstr 7014.
80. van Meerbeeck JP, Kramer GW, Van Schil PE, et al. Randomized controlled trial of resection versus radiotherapy after induction chemotherapy in stage IIIA-N2 non-small-cell lung cancer. J Natl Cancer Inst 2007;99:442-50.
81. Thomas M, Rübe C, Hoffknecht P, et al. Effect of preoperative chemoradiation in addition to preoperative chemotherapy: a randomised trial in stage III non-small-cell lung cancer. Lancet Oncol 2008;9:636-48.
82. Koshy M, Fedewa SA, Malik R, et al. Improved survival associated with neoadjuvant chemoradiation in patients with clinical stage IIIA(N2) non-small-cell lung cancer. J Thorac Oncol 2013;8:915-22.
83. Pataer A, Kalhor N, Correa AM, et al. Histopathologic response criteria predict survival of patients with resected lung cancer after neoadjuvant chemotherapy. J Thorac Oncol 2012;7:825-32.
84. Grossi F, Rijavec E, Barletta G, et al. A multicenter phase II randomized study of customized neoadjuvant therapy versus standard chemotherapy (CT) in non-small cell lung cancer (NSCLC) patients with resectable stage IIIA (N2) disease (CONTEST trial). J Clin Oncol 2013;31:abstr TPS7606.
85. Tada H, Takeda K, Nakagawa K, et al. Vinorelbine plus cisplatin versus gefitinib in resected non-small cell lung cancer haboring activating EGFR mutation (WJOG6410L). J Clin Oncol 2012;30:abstr TPS7110.

**Cite this article as:** Uramoto H, Tanaka F. Recurrence after surgery in patients with NSCLC. Transl Lung Cancer Res 2014;3(4):242-249. doi: 10.3978/j.issn.2218-6751.2013.12.05

© Translational lung cancer research. All rights reserved.
